# Editorial: Innovation, technology and sustainability in sport management and education

**DOI:** 10.3389/fspor.2026.1880677

**Published:** 2026-06-05

**Authors:** Liandi Van Den Berg, Brendon Knott, Rakesh Tomar, Karen Danylchuk, Ruth Crabtree

**Affiliations:** 1 School of Management Sciences, Faculty of Economic and Management Sciences, North-West University, Potchefstroom, South Africa; 2 Sport Management Department, Cape Peninsula University of Technology, Cape Town, South Africa; 3 Department of Physical Education, King Fahd University of Petroleum & Minerals, Dhahran, Saudi Arabia; 4 School of Kinesiology, Faculty of Health Sciences, Western University, London, ON, Canada; 5 School of Sport & Physical Activity, Sheffield Hallam University, Sheffield, United Kingdom

**Keywords:** education, innovation, management, sport, sustainability, technology

The contemporary sport ecosystem is undergoing a profound transformation driven by rapid technological innovation, rising societal expectations for sustainability, and evolving educational paradigms. Across professional sport, grassroots participation, school sport, esports, and sport management education, stakeholders are increasingly required to navigate the intersections between digital advancement, ethical responsibility, institutional sustainability, and human development.

The collection of papers in this Research Topic reflects the multidimensional and interdisciplinary nature of contemporary sport management and education research. Collectively, the contributions span diverse geographical contexts, including South Africa, China, Türkiye, Indonesia, and Central and Eastern Europe, while addressing issues ranging from digital transformation and esports governance to school sport policy, competitive advantage theory, and social equity in sport education. Together, these studies illustrate how innovation and sustainability are no longer isolated concepts but interconnected drivers shaping the future of sport participation, governance, education, and organisational development.

A central theme emerging from this collection is the role of technology as both an enabler and a disruptor within sport systems. The article by Wang, *Exploration of the Path of Digital Technology Empowering the Sustainable Development of Alumni Football*, demonstrates how digital technologies support the sustainable development of grassroots and amateur sport structures. Using ordinal logistic regression, this study analysed survey data from university football alumni to examine 10 digital factors influencing development. The findings show that digital business models and digital team culture play a significant role in sustainability, whereas virtual coaching and match data management have a lesser impact. The study offers practical insights for policymakers, educators, and industry stakeholders seeking to enhance participation, digital inclusion, and sustainable growth in sport.

Similarly, the growing influence of digitalisation is evident in the contribution by Hospodková et al., *Searching Synergy Between Esports and Academia: The Role of Sport Faculties and Departments in CEE Countries*. Through a qualitative approach, the research found that universities can play a significant role in the development and professionalisation of esports through collaboration, education, and research. Findings indicated that universities can support esports teams and players by conducting research on player health and performance, offering specialised courses and certifications for coaches and managers, and facilitating access to experts in nutrition and sleep. The study also found that universities can help establish industry standards and licensing requirements in partnership with esports associations. Overall, the research emphasised the importance of collaboration between universities and esports organisations, as well as the need for innovation, formal training programmes, and stronger promotion of esports within the university environment.

Innovation in sport management is also examined from a theoretical and strategic perspective in the article by Riegler and Daumann, “*Transferability of the First-Mover Advantage to Individual Sports: A Conceptual and Theoretical Analysis*.” The authors extend the concept of first-mover advantage, traditionally associated with business and economics, to the context of national sports associations in individual sports. Through comparative analysis, theoretical modelling, and case logic, the study demonstrates the applicability of strategic management theories to sport organisations. This contribution is significant because it highlights the growing sophistication of sport management research and the increasing relevance of strategic innovation within competitive sport governance. By conceptualising how national associations may benefit from pioneering strategies, the article provides a foundation for future empirical work exploring innovation-led competitive advantage in sport systems.

Sustainability and social responsibility emerge as another critical dimension. In *The Goals and Selection Criteria of Sports Scholarships: Mutual Benefits Perceived by South African Schools*, Ekron et al. investigate sports scholarship systems within South African elite schools through the lens of Social Exchange Theory. The study reveals the complex balance between institutional competitiveness, athlete development, diversity, and equitable access within scholarship allocation processes. The findings demonstrate that sustainability in sport management encompasses social sustainability, inclusion, and ethical governance. Particularly within the South African context, where historical inequalities continue to shape educational opportunities, the article provides important insights into how sport systems may simultaneously reproduce and challenge social disparities.

Educational sustainability and policy development are further explored in Ma'mun et al.'s study, *Physical Education and School Sport in Emerging Nations: A Comparison of Indonesia and Türkiye*. The study compares physical education curricula, school sport structures, extracurricular provision, and institutional resources across the two countries. The findings reveal shared challenges relating to infrastructure, staffing, and integration of schools and clubs. However, the study also highlights opportunities to strengthen school sport systems by investing in facilities, enhancing extracurricular programming, and strengthening community partnerships. The article reinforces the notion that sustainable sport development begins within educational systems and requires coordinated policy support, institutional commitment, and long-term strategic planning.

Collectively, these contributions feature several broader trends shaping the future of sport management and education. Digital transformation is central to all levels of sport, from grassroots participation and esports to organisational strategy and educational delivery. In addition, the research indicates that sustainability must be understood holistically, encompassing environmental responsibility, social inclusion, institutional resilience, and ethical governance. In this regard, sport education institutions are critical actors in facilitating innovation, developing future leaders, and promoting interdisciplinary collaboration. The collection also highlights the importance of contextual diversity in sport management research. The studies highlight how innovation and sustainability manifest differently across cultural, economic, and institutional environments, demonstrating that localised challenges require context-sensitive solutions. From a methodological perspective, the Research Topic showcases a diversity of approaches, including qualitative inquiry, conceptual analysis, comparative research, and quantitative modelling. Such methodological plurality is essential for addressing the complexity of contemporary sport systems and reflects the interdisciplinary character of sport management and education research.

As Guest Editors, this collection contributes meaningfully to ongoing scholarly conversations regarding the future of sport in a rapidly changing world. We hope that the contributions presented here stimulate further interdisciplinary research, encourage international collaboration, and inspire both scholars and practitioners to continue exploring how sport can contribute positively to social, educational, technological, and sustainable development.

